# Mapping the research landscape of virtual reality and artificial intelligence in medical education evaluation: A bibliometric analysis

**DOI:** 10.1097/MD.0000000000050003

**Published:** 2026-08-07

**Authors:** Xi Huang, Xingxin Li, Qianwei Lu, Zhengjun Guo, Na Wang, Yifan Zhang, Yanni Hu, Di Li, Yun Luo, Xia Wu

**Affiliations:** a Department of Hematology, The Second Affiliated Hospital of Chongqing Medical University, Chongqing, China; b Experimental Teaching Center, Chongqing Medical University, Chongqing, China; c Department of Oncology and Hematology, The Beibei Affiliated Hospital of Chongqing Medical University, Chongqing, China; d Department of Cancer Center, The Second Affiliated Hospital of Chongqing Medical University, Chongqing, China; e Educational Administration Office, The Second Affiliated Hospital of Chongqing Medical University, Chongqing, China; f Department of Hematology, Children’s Hospital of Chongqing Medical University, Chongqing, China; g Department of Pharmacy, The Second Affiliated Hospital of Chongqing Medical University, Chongqing, China.

**Keywords:** artificial intelligence, bibliometric analysis, competency assessment, medical education evaluation, virtual reality

## Abstract

**Background:**

Evaluation of medical education is essential for ensuring the quality of health professional training. However, conventional evaluation approaches often lack objectivity, scalability, and longitudinal assessment capacity. Virtual reality (VR) and artificial intelligence (AI) are increasingly integrated into medical education, yet their application in educational evaluation has not been systematically characterized.

**Objective:**

To examine research trends, thematic evolution, and emerging directions in VR- and AI-enabled medical education evaluation, a bibliometric analysis was conducted.

**Methods:**

Publications indexed in the Web of Science Core Collection between January 1, 2015, and December 31, 2025, were retrieved using predefined search terms related to VR, AI, medical education, and evaluation. Eligible English-language articles and reviews were analyzed using CiteSpace (version 6.4.R2). Annual publication and citation trends, country collaboration patterns, and cited journals were assessed. Research themes and frontiers were examined through keyword co-occurrence, clustering, burst detection, and timeline analyses.

**Results:**

A total of 695 publications were included. Annual publications and citations increased steadily, with accelerated growth after 2020. The United States, Germany, China, England, and Canada produced the highest number of publications, whereas Belgium, Egypt, Sweden, Singapore, and Switzerland demonstrated high collaboration centrality. Influential cited journals were concentrated in medical education and simulation-based training domains. Keyword analyses identified major themes including surgical education, VR simulation, clinical reasoning, decision support, and residency and undergraduate education. Burst and timeline analyses indicated a progression from early simulation-based skill validation toward learner-centered performance evaluation and, more recently, quality-oriented and curriculum-level assessment.

**Conclusions:**

Research on VR- and AI-enabled medical education evaluation has expanded rapidly and evolved from technical skill assessment toward comprehensive, competency-oriented, and quality-focused evaluation. These findings highlight the growing role of emerging technologies in shaping future global medical education evaluation frameworks.

## 1. Introduction

The integration of virtual reality (VR) and artificial intelligence (AI) into medical education represents a transformative shift in how learner performance is assessed and supported. VR-based simulations provide standardized, immersive environments for evaluating both technical and non-technical skills, while AI-driven systems now enable automated scoring, personalized feedback, and competency tracking. Recent studies demonstrate the feasibility of AI-powered formative feedback systems in undergraduate medical education, with high levels of learner and mentor acceptance,^[1]^ as well as measurable improvements in structured, rubric-based feedback supported by generative AI.^[2]^ In simulation contexts, AI-assisted assessment of non-technical skills shows partial agreement with expert raters, indicating potential for hybrid human–AI evaluation models.^[3]^ Immersive technologies are increasingly incorporated into patient safety and anesthesiology training to enhance competency-based assessment frameworks.^[4]^

At the same time, concerns remain regarding the validity, reliability, and safety of AI-supported assessment. Evaluations of large language models on medical examination-style questions reveal strong performance in text-based tasks but reduced reliability in image-based interpretation, raising important questions about assessment robustness.^[5]^ Similarly, while AI-based chatbots providing real-time communication feedback demonstrate high perceived accuracy and usefulness, further validation is required before broad curricular integration.^[6]^ These findings underscore both the promise and the methodological challenges associated with integrating VR and AI into high-stakes educational evaluation.

Although publications on VR- and AI-enabled medical education have increased markedly in recent years, the intellectual structure and developmental trajectory of research specifically focused on educational evaluation remain unclear. Existing studies are often technology- or specialty-specific and lack a comprehensive synthesis of thematic evolution and research frontiers. Therefore, this study conducted a bibliometric analysis to map publication patterns, collaboration networks, and knowledge structures in VR- and AI-based medical education evaluation. By delineating the temporal and thematic dynamics of this emerging field, the study aims to provide an evidence-based foundation for future research and the development of more scientific and standardized evaluation frameworks.

## 2. Material and methods

### 2.1. Data source and search strategy

This bibliometric study was conducted using the Web of Science Core Collection (WoSCC) as the primary data source. The search was limited to English-language publications. The retrieval strategy was as follows: TS = (virtual reality OR artificial intelligence) AND (education OR teaching) AND (medicine OR medical) AND (assessment OR evaluation). The data retrieval was performed on January 6, 2026. Publications were included if they met the following criteria: the topic involved the application of VR or AI in medical education and educational evaluation; the document type was an original research article or review; the publication language was English; the publication date ranged from January 1, 2015, to December 31, 2025.

Exclusion criteria were as follows: conference abstracts, editorial materials, news items, and book chapters; duplicate records; publications irrelevant to the research topic; records with incomplete bibliographic information that prevented content analysis.

**Figure 1.**
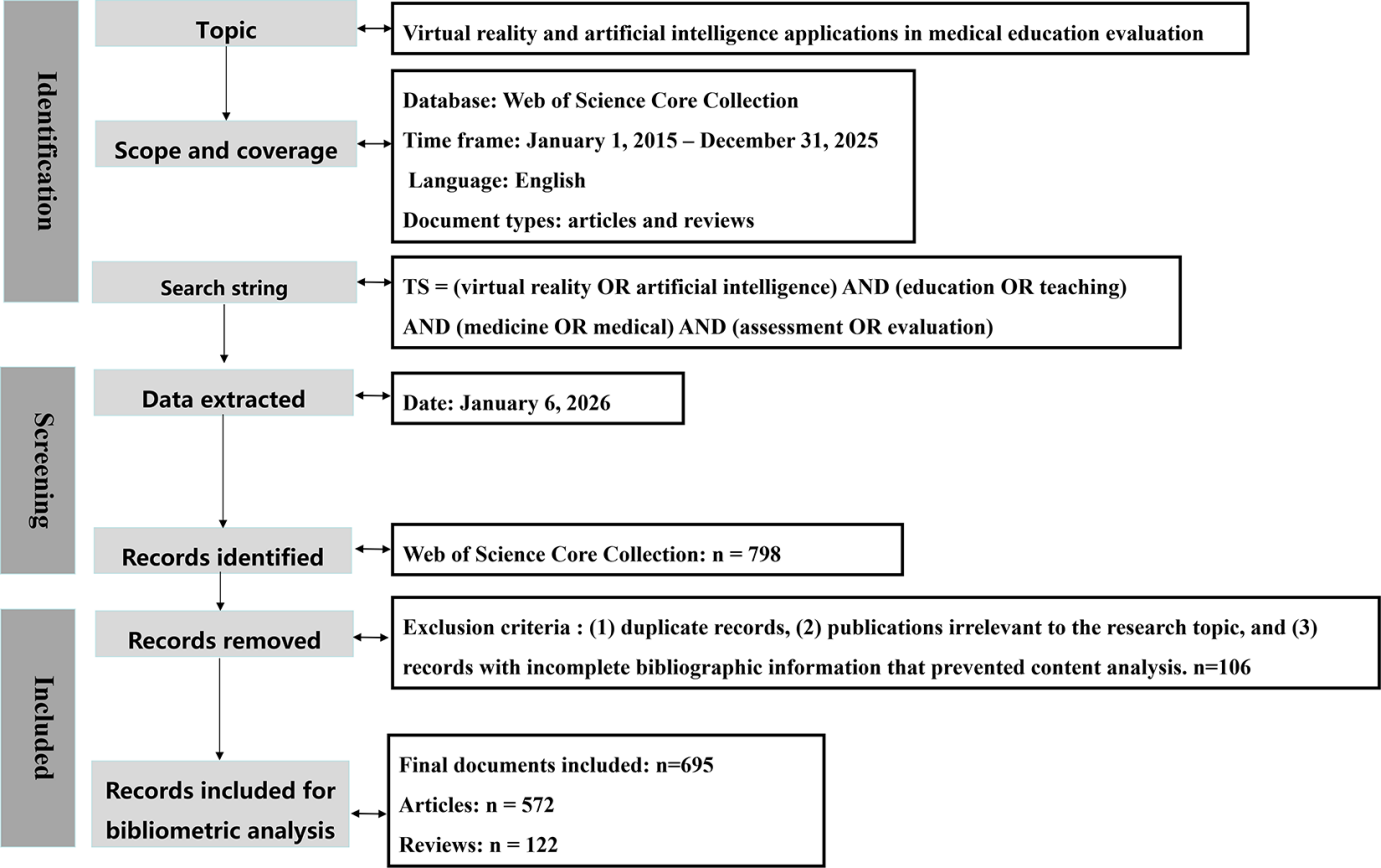
Flow diagram of included studies. n = number of records, TS = test statistic.

After screening, a total of 695 publications were included in the final analysis (Fig. 1) .

### 2.2. Study selection and data extraction

Two researchers independently screened the retrieved records. Titles and abstracts were first reviewed to remove duplicates and irrelevant publications. In cases of disagreement, consensus was reached through discussion. Full records of the included publications were exported in text format, and bibliographic information (including title, authors, publication year, country, journal, references, and keyword) was extracted for subsequent analysis.

### 2.3. Bibliometric analysis and visualization

Bibliometric and visualized analyses were conducted using CiteSpace (version 6.4.R2). The software was employed to explore publication trends, citation patterns, international collaboration networks, influential journals, and the thematic evolution of research on VR and AI in medical education evaluation.

The time span was set from January 1, 2015, to December 31, 2025, with a time slice of 1 year. The node types included country, cited journal, and keyword, corresponding to analyses of global collaboration, knowledge sources, and research hotspots. To enhance network clarity and reduce visual complexity, Pathfinder, pruning sliced networks, and pruning the merged network were applied.

Keyword co-occurrence, clustering, burst detection, and timeline analyses were performed to identify research themes, emerging topics, and their temporal evolution. Network maps were interpreted based on frequency, betweenness centrality, and temporal distribution to reveal the intellectual structure and development trajectory of the field.

### 2.4. Quality control and interpretation strategy

As this study focused on bibliometric and visualization analysis rather than empirical outcome synthesis, no formal quality appraisal of individual studies was conducted. Instead, greater interpretive emphasis was placed on highly cited publications, core journals, and structurally important nodes within the networks, which were considered representative of influential research in the field.

### 2.5. Ethical considerations

This study was based exclusively on bibliographic data retrieved from publicly available, peer-reviewed literature and did not involve human participants, animal subjects, clinical samples, or identifiable personal information. Therefore, informed consent was not required. Ethical approval was waived by the institutional ethics committee of The Second Affiliated Hospital of Chongqing Medical University, as the study involved secondary analysis of published data only. Patients, animals, or members of the public were not involved in the study design, data analysis, interpretation, reporting, or dissemination of the findings.

## 3. Result

### 3.1. Development trends of research on VR and AI in medical education evaluation

**Figure 2.**
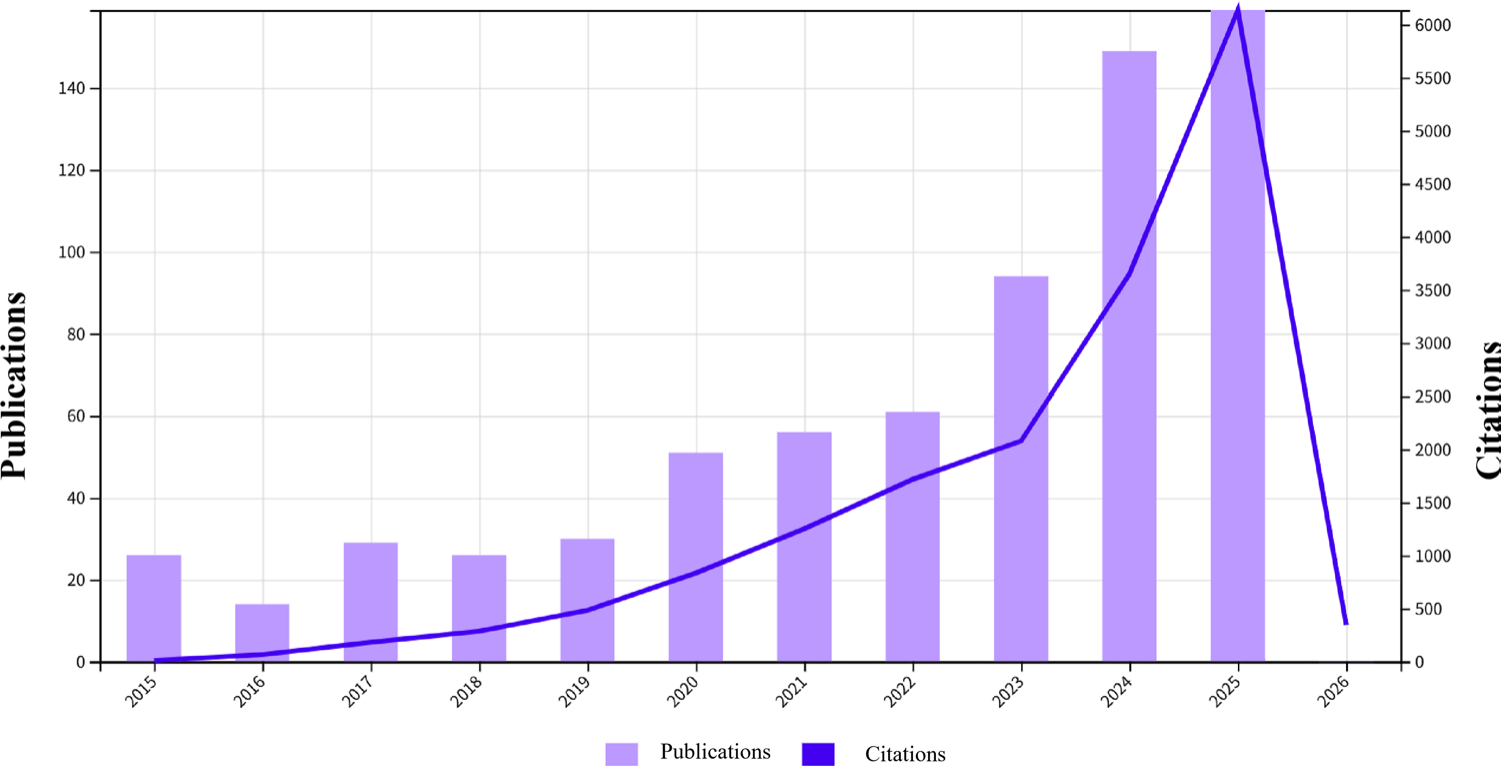
Trends of publication and citation numbers.

To examine the temporal evolution of research output, annual publication and citation trends related to VR and AI in medical education evaluation were analyzed. Figure 2 shows a steady increase in the number of publications over the past decade, indicating sustained growth in scholarly interest. In particular, a noticeable acceleration in publication output has occurred since 2020, suggesting an expanding application of VR and AI technologies in evaluation-oriented medical education research.

To further assess academic impact, citation trends were examined over time. The citation curve remained relatively stable before 2019, followed by a steeper increase between 2019 and 2023. After 2023, citations rose more sharply, reaching a peak in 2025. This pattern indicates not only increasing research output but also growing recognition and influence of studies focusing on VR- and AI-based medical education evaluation.

### 3.2. Influential journals, countries in VR- and AI-based medical education evaluation

The structural foundations and collaborative landscape of research on VR and AI-enabled evaluation in medical education were characterized through country collaboration and cited journal analyses.

**Figure 3.**
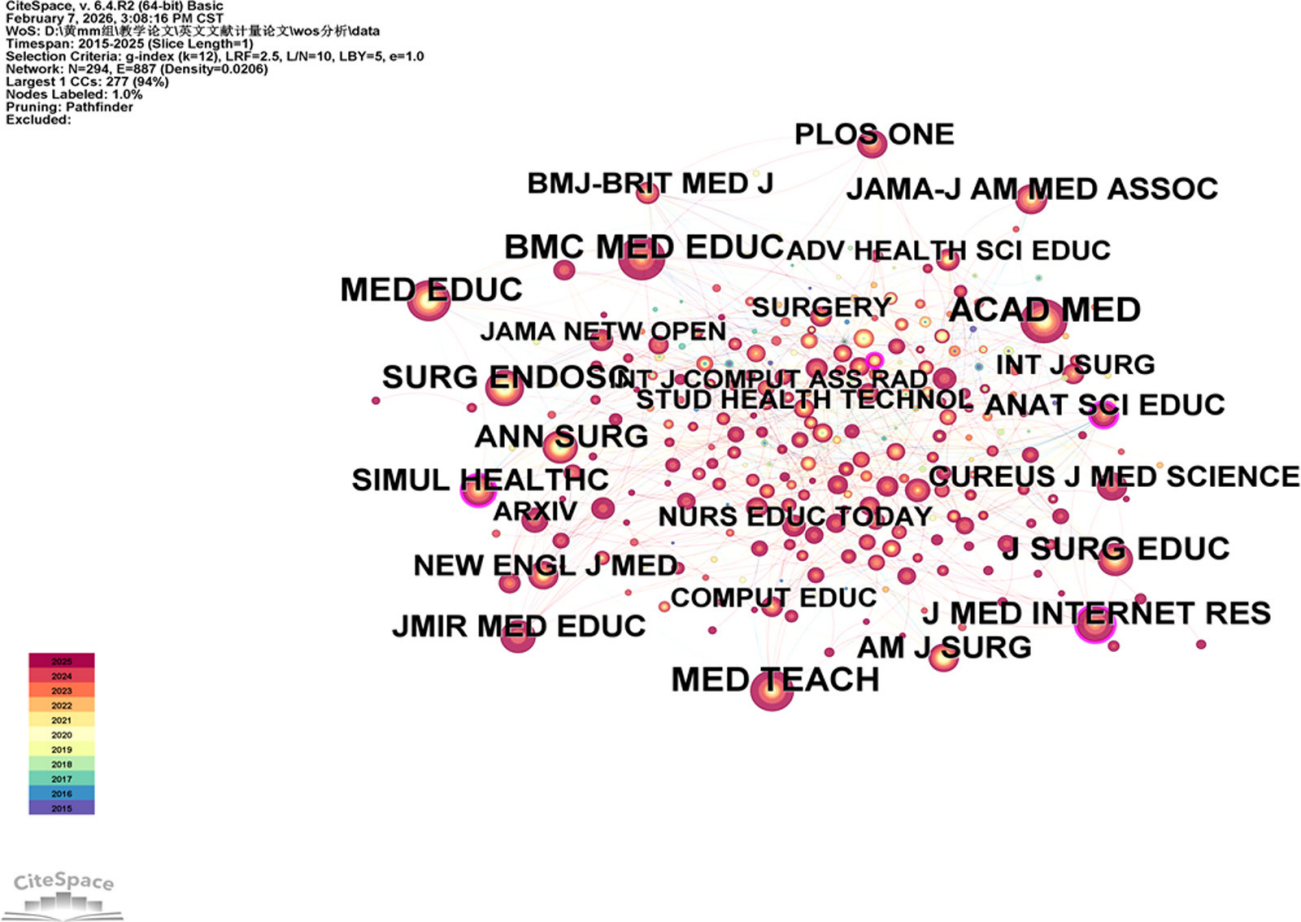
Cited journal co-citation network. Node size represents citation frequency, and line thickness indicates co-citation strength between journals.

A country collaboration network showed that the United States, Germany, China, England, and Canada contributed the highest number of publications. In contrast, countries such as Belgium, Egypt, Sweden, Singapore, and Switzerland exhibited high betweenness centrality, suggesting that they play important bridging roles in international collaboration (Table 1). Overall, the collaboration network reflects a geographically diverse research landscape, with both high-output countries and high-centrality countries jointly shaping the development of this field (Fig. 3).

**Table 1:** Top 10 countries of publications and centrality.

Category	Rank	Country	Documents	Centrality
High productivity	1	USA	184	0.37
	2	CHINA	113	0.2
	3	GERMANY	91	0.14
	4	CANADA	71	0.07
	5	ENGLAND	67	0.11
	6	AUSTRALIA	36	0.11
	7	DENMARK	36	0.01
	8	FRANCE	30	0.06
	9	SWITZERLAND	25	0.11
	10	NETHERLANDS	25	0.06
High centrality	1	USA	184	0.37
	2	BELGIUM	9	0.21
	3	CHINA	113	0.2
	4	EGYPT	6	0.17
	5	GERMANY	91	0.14
	6	ENGLAND	67	0.11
	7	AUSTRALIA	36	0.11
	8	SWITZERLAND	25	0.11
	9	GREECE	9	0.11
	10	POLAND	7	0.11

USA = United States of America.

**Figure 4.**
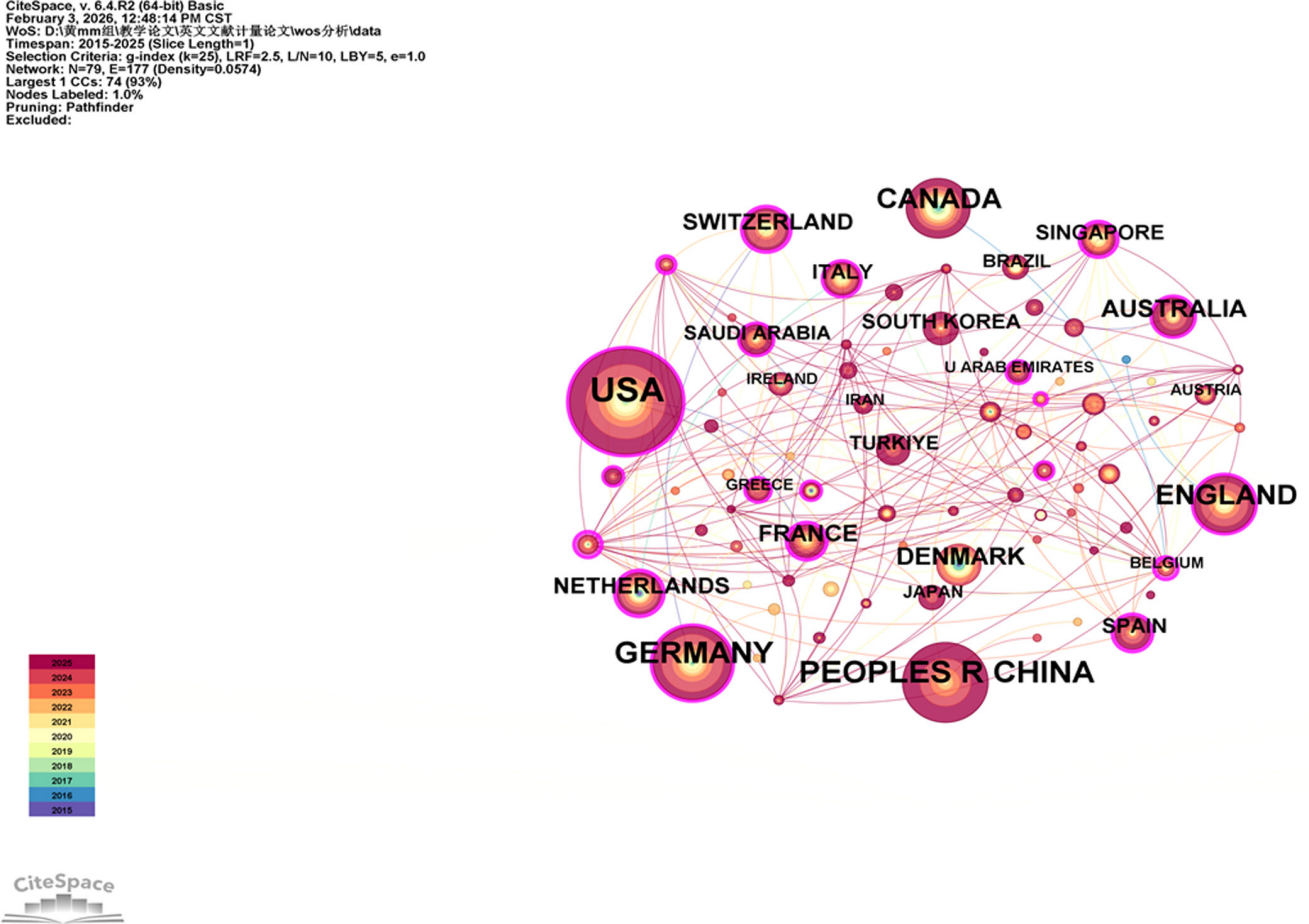
Country collaboration network. Node size reflects total publications, and line thickness represents collaboration strength between countries.

Cited journal analysis further revealed that research on VR and AI-based medical education evaluation is primarily disseminated through a group of core journals spanning medical education, simulation-based training, surgery, and digital health. Journals such as Academic Medicine, Medical Education, BMC Medical Education, and the Journal of Medical Internet Research demonstrated high citation frequencies, indicating their central role in shaping methodological standards and research directions. The prominence of both traditional medical education journals and technology-oriented journals highlights the interdisciplinary characteristics of the field and reflects the integration of educational theory, clinical training, and emerging digital technologies (Fig. 4).

### 3.3. Identification of research hot spots using keyword analysis

**Figure 5.**
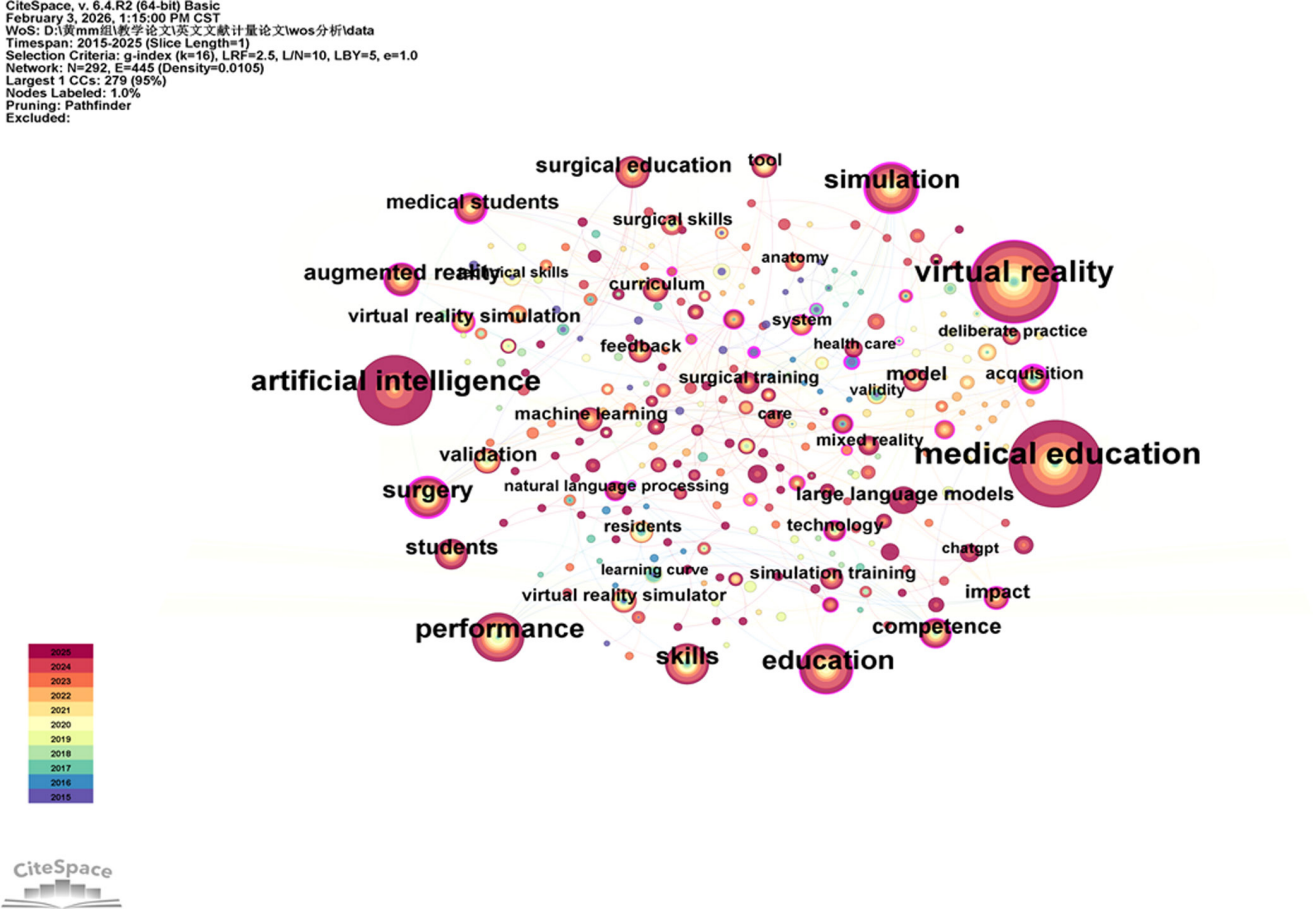
Keyword co-occurrence network. Node size corresponds to keyword frequency, and node color represents the average occurrence year (purple represents older terms; red indicates more recent terms).

To identify core research themes, a keyword co-occurrence network was generated (Fig. 5). High-frequency keywords included medical education, VR, simulation, education, and performance, indicating that simulation-based educational contexts formed the foundation of evaluation research.

Keywords with high betweenness centrality, such as acquisition, surgery, simulation, and medical students, occupied critical positions within the network, highlighting the central role of skill acquisition, surgical training, and learner performance evaluation. AI-related keywords, including AI, machine learning, natural language processing, large language models, and ChatGPT, appeared later but demonstrated increasing connectivity, reflecting the gradual integration of AI-driven methods into evaluation practices.

**Figure 6.**
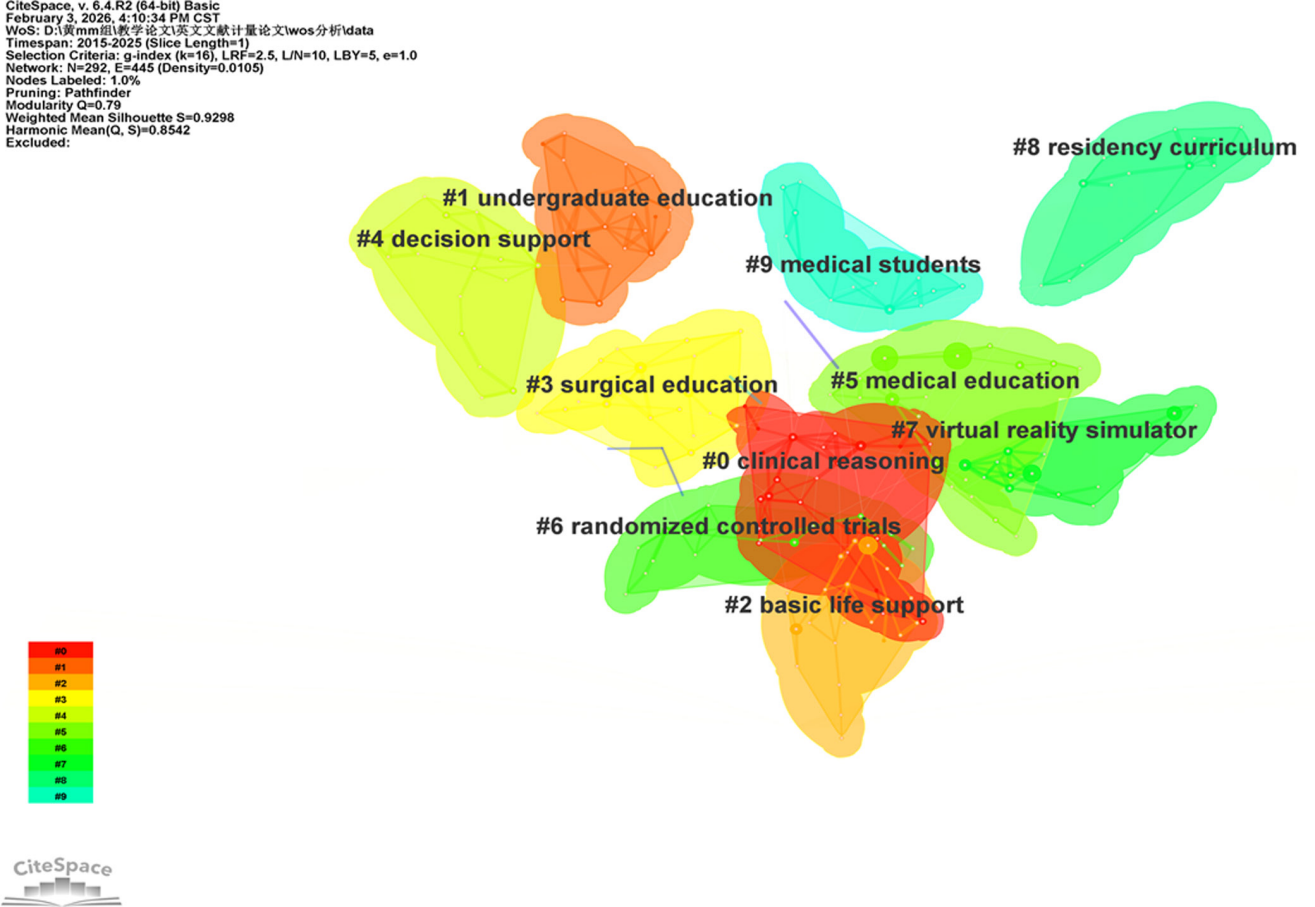
Keyword cluster view. Different colors represent distinct keyword clusters, and links indicate co-occurrence relationships between keywords. Cluster labels denote the thematic categories identified by the clustering analysis.

To explore the thematic structure of the field, keyword clustering analysis identified several major research clusters (Fig. 6). Early and technology-oriented clusters included #3 Surgical education, #7 VR simulation, and #2 Basic life support, emphasizing VR-based simulation and procedural skill assessment. Educational context-focused clusters, such as #5 Medical education, #9 Medical student, #8 Residency curriculum, and #1 Undergraduate education, reflect the application of evaluation frameworks across different stages of medical training.

More conceptually oriented clusters, including #0 Clinical reasoning and #4 Decision support, suggest a growing emphasis on higher-order cognitive evaluation and AI-assisted decision-making processes. The presence of #6 Randomized controlled trials highlights increasing methodological rigor and validation efforts within the field.

**Figure 7.**
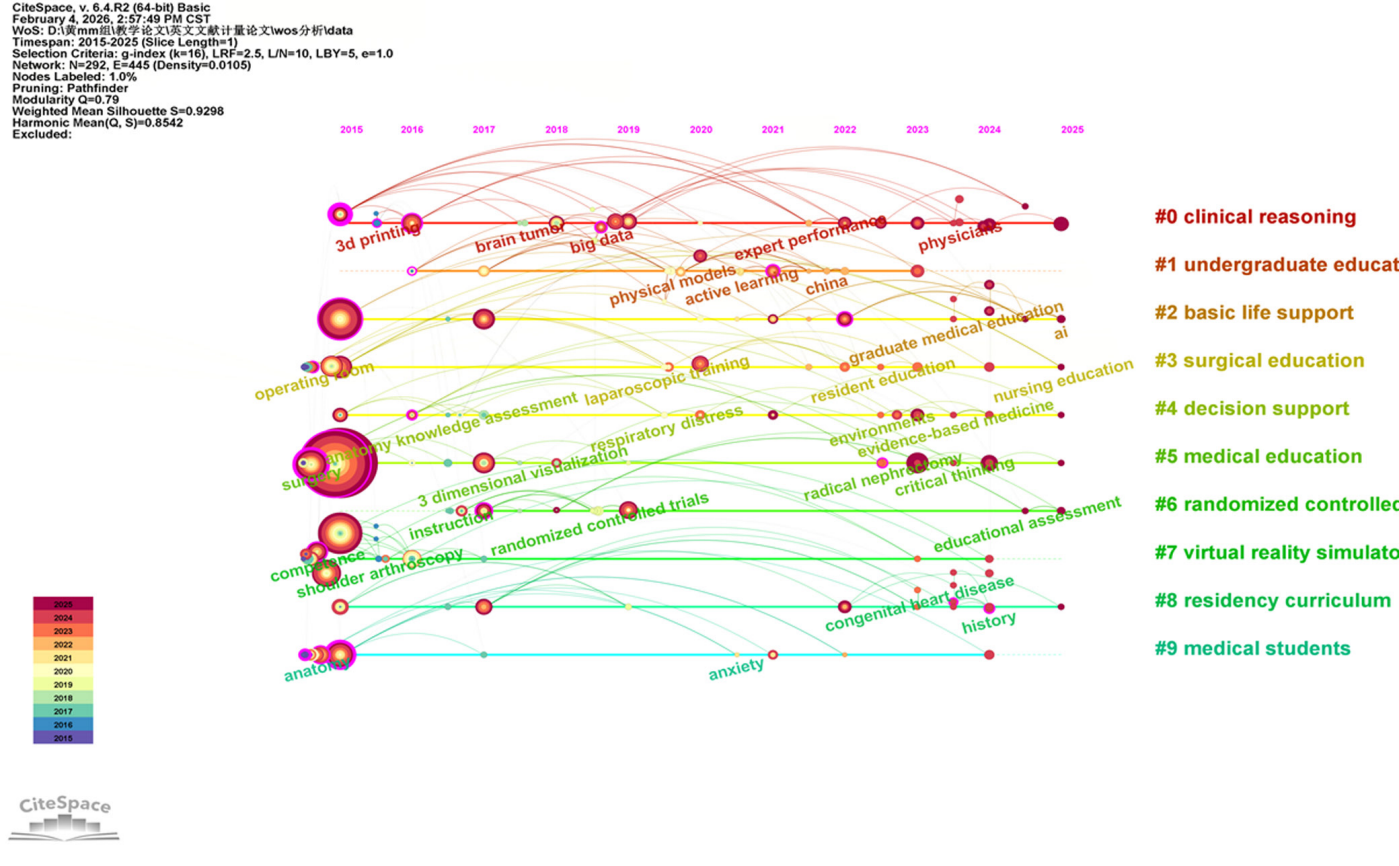
Keyword citation burst analysis illustrating burst strength and temporal span (initial to final year) of emerging keywords.

To identify research hot spots over time, a keyword burst detection analysis was conducted (Fig. 7). Early burst terms between 2015 and 2019, including learning curve, validity, competence, technical skills, and VR simulation, indicate an initial focus on skill acquisition and the validation of simulation-based assessment tools. Subsequent bursts from 2017 to 2021, such as residents, performance, validation, and proficiency, reflect increasing attention to learner-centered evaluation and training effectiveness across medical education levels, alongside emerging interest in curriculum-based educational tools. More recent bursts after 2020 shifted toward undergraduate education and quality, with quality remaining active from 2023 to 2025, suggesting a growing emphasis on evaluation quality and standardization. Concurrently, AI-related keywords showed increasing burst strength, highlighting the expanding role of advanced computational approaches in medical education assessment. Overall, burst analysis demonstrates a progression from early simulation-based validation toward curriculum-level, quality-oriented, and AI-enhanced evaluation research.

**Figure 8.**
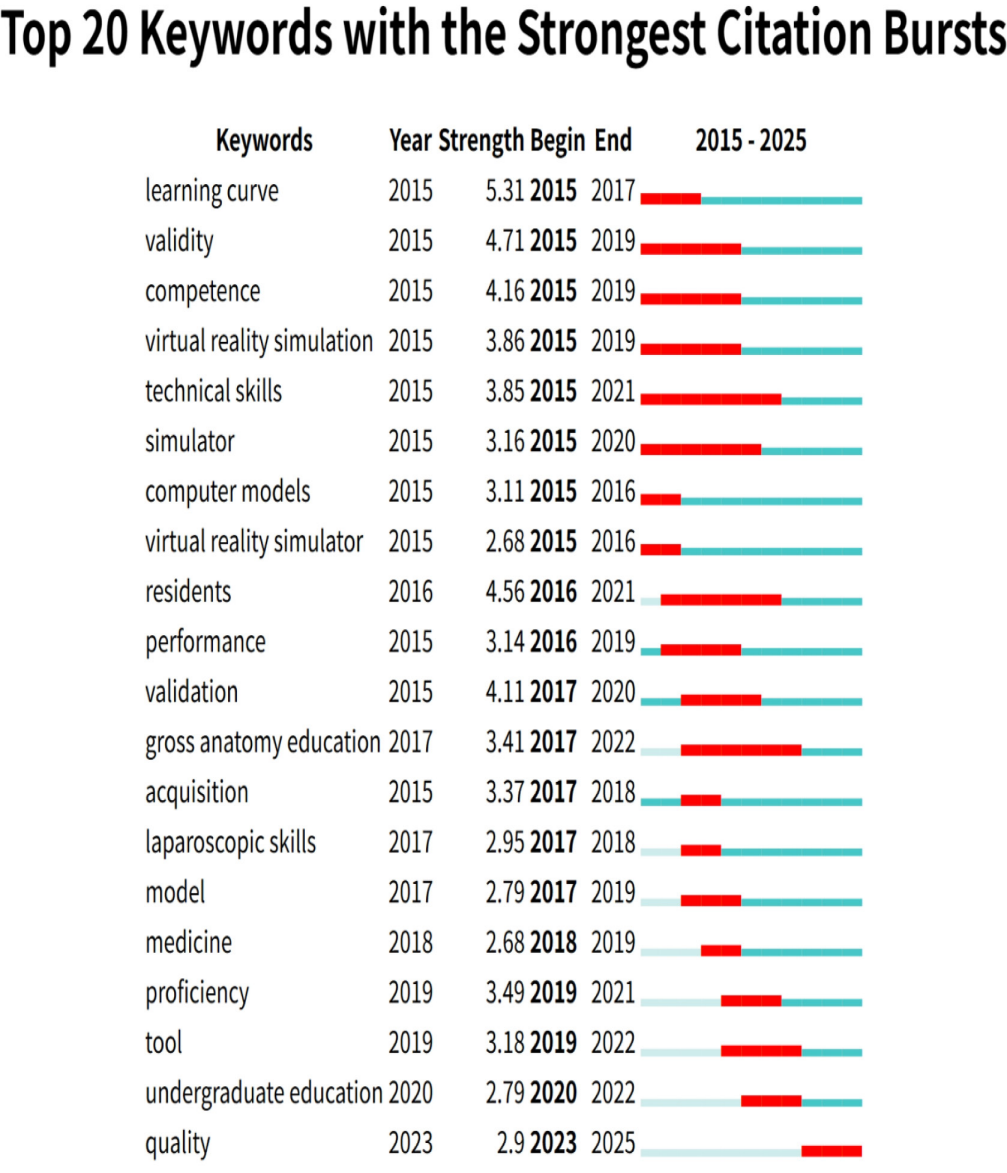
Keyword timeline view. Horizontal positions represent the temporal distribution of keyword clusters, and lines indicate the duration of each cluster over time. Cluster labels denote the thematic categories identified by the timeline analysis.

To illustrate the temporal evolution of research themes in VR- and AI-based medical education evaluation, a keyword timeline map was generated (Fig. 8). Early activity was dominated by clusters such as Surgical education, Medical education, VR simulation, and Medical student, indicating an initial emphasis on procedural skill assessment and simulation-based training contexts. Subsequent clusters, including Clinical reasoning, Basic life support, and Decision support, reflect a shift toward cognitive evaluation, emergency training assessment, and technology-assisted decision-making. More recent themes, such as Residency curriculum, Undergraduate education, and Randomized controlled trials, suggest growing attention to curriculum-level evaluation and evidence-based research designs. Several clusters, particularly Surgical education, Clinical reasoning, and Medical education, persisted throughout the study period, indicating sustained research interest. Collectively, the timeline map reveals a progression from early simulation-centered skill evaluation toward comprehensive, curriculum-oriented, and methodologically rigorous evaluation frameworks.

## 4. Discussion

### 4.1. Interpretation of research evolution in medical education evaluation

This bibliometric analysis demonstrates the progressive integration of VR and AI into medical education evaluation. Rather than representing a discrete technological shift, the observed evolution reflects a broader transformation in how competence, educational quality, and accountability are conceptualized within global medical training systems.^[7]^ The sustained increase in publications and citations identified in the Results indicates that technology-enabled evaluation has moved from exploratory experimentation to an established research trajectory.^[8,9]^

The accelerated growth after 2020 appears to reflect both technological maturation and systemic pressures within health professions education. The global adoption of competency-based medical education has intensified demand for objective, scalable, and longitudinal assessment strategies.^[10,11]^ VR-based simulation offers standardized, reproducible performance environments,^[12,13]^ while AI-driven analytics enables automated scoring and large-scale data processing.^[14]^ The temporal surge in research output coincides with widespread shifts toward digital and simulation-based education, which may suggest that technology-mediated evaluation potentially contributed to a stabilizing role during disruptions to traditional clinical training.^[15]^ Therefore, the literature indicates that VR and AI have emerged not only as innovative tools but also as structural supports for educational continuity and quality assurance.

Thematic evolution further clarifies this trajectory. Early research clusters centered on simulation feasibility and procedural skill measurement, consistent with the validation phase of immersive technologies.^[16,17]^ Over time, keyword timelines reveal increasing emphasis on clinical reasoning, cognitive processes, and competence-oriented assessment.^[9,18]^ This observed shift appears to reflect a movement from task-based measurement toward integrated evaluation of decision-making and authentic performance. Such progression mirrors broader developments in medical education scholarship, which increasingly prioritizes contextualized competence over isolated technical proficiency.

### 4.2. Transition from tool validation to quality-oriented evaluation

A prominent bibliometric finding was the growing visibility of terms related to validation, educational outcomes, and quality assurance.^[19]^ This finding is consistent with the possibility that the field is transitioning from proof-of-concept validation toward systematic evaluation of educational impact. Citation bursts associated with outcome measurement and program evaluation reinforce this interpretation, indicating methodological maturation.^[20]^

The emergence and persistence of undergraduate and residency curriculum clusters further illustrate this shift.^[21,22]^ Rather than examining VR or AI as standalone interventions, recent research situates these technologies within structured curricular pathways and competency frameworks. The timeline persistence of curriculum-related clusters suggests institutional embedding rather than isolated experimentation.^[8,23]^ This trend reflects a recognition that technological innovation alone does not guarantee educational benefit; meaningful impact depends on alignment with curricular design, accreditation standards, and assessment infrastructure.

Collectively, these findings suggest that the sustainable adoption of VR- and AI-based evaluation requires integration within institutional assessment strategies. The bibliometric patterns indicate that quality-oriented frameworks increasingly shape both research priorities and implementation strategies. As technologies become embedded within curricula, evaluation research correspondingly shifts toward examining reliability, validity, and outcome relevance at the program level.

### 4.3. Expanding role of AI in educational assessment

Burst analysis revealed the increasing prominence of AI-related concepts such as decision support, predictive modeling, and performance evaluation.^[24]^ This pattern may signal a methodological shift in educational assessment: AI is evolving from a supportive instructional adjunct to an analytical infrastructure capable of longitudinal competency tracking and adaptive feedback generation.

From a systems perspective, AI-supported evaluation addresses persistent challenges in medical education assessment, including inter-rater variability, scalability limitations, and fragmentation of learner performance data across training stages.^[14]^ When combined with VR-based simulation environments, AI enables continuous capture and analysis of multimodal performance metrics, facilitating data-driven feedback and longitudinal monitoring.^[25,26]^ The results indicate that such integration represents a growing research frontier, with clusters increasingly centered on analytics-driven evaluation models.

However, the bibliometric trends also reveal parallel attention to issues of transparency, interpretability, and cross-context validity.^[27,28]^ As AI-mediated assessment expands across institutions and geographic regions, concerns regarding fairness, algorithmic bias, and generalizability become more prominent.^[29,30]^ Recent clusters demonstrate heightened interest in validation frameworks and ethical considerations, indicating that methodological scrutiny is developing alongside technological innovation.^[31,32]^ Ensuring robustness across curricular contexts and learner populations remains critical for broader adoption.

### 4.4. Implications for future research and educational practice

The collaboration networks and thematic trajectories identified in this study suggest that future research should prioritize integrative evaluation models that combine VR and AI technologies with pedagogical theory and competency frameworks. The results demonstrate increasing international collaboration, indicating that cross-institutional partnerships may facilitate shared standards and interoperable assessment systems.^[8]^

Future investigations should move beyond isolated validation studies toward multisite, longitudinal research examining reliability, validity, and educational outcomes across diverse learner populations.^[33]^ Comparative studies across undergraduate and residency contexts may clarify how VR- and AI-based evaluation functions at different stages of professional development.^[22]^ Additionally, standardized reporting frameworks and shared performance metrics could enhance reproducibility and cross-context generalizability.

From a practical perspective, educators and policymakers should adopt VR- and AI-based evaluation tools that are explicitly aligned with competency standards and curricular pathways.^[11]^ As reflected in the curriculum-level clusters identified in this study, the integration of these tools within structured programs may enhance both assessment reliability and educational coherence. Achieving this integration will require sustained interdisciplinary collaboration among clinicians, educators, and data scientists to ensure that emerging systems remain technically rigorous, pedagogically grounded, and adaptable across diverse educational environments.^[31,32]^

## 5. Advantages and limitations of research

Previous bibliometric studies have examined VR and AI in medical education primarily from an instructional perspective, with emphasis on simulation training and technology-enhanced learning environments.^[8,9]^ Several reviews have noted that most research has historically evaluated immersive technologies as teaching tools rather than as components of formal assessment systems.^[15]^

In contrast, the present study centers specifically on medical education evaluation, a dimension comparatively underrepresented in earlier bibliometric mappings.^[34]^ By delineating evaluation-related constructs such as validity, performance, competence, and quality, our analysis demonstrates how emerging technologies are increasingly embedded within competency assessment and curriculum-level evaluation frameworks.^[11,20]^

Our findings further indicate a shift from early simulation-based skill validation toward quality-oriented and program-integrated assessment across undergraduate and residency education.^[22,29]^ This transition aligns with recent scholarship suggesting that VR- and AI-enabled tools are reshaping assessment paradigms and enabling longitudinal competency tracking across training stages.^[35]^ From a broader perspective, the results extend prior bibliometric work by showing that VR and AI are not only transforming instructional strategies but are also redefining the global paradigm of medical education evaluation.^[7]^

It is imperative to acknowledge the limitations of the present study. The analysis relied exclusively on a single database (WoSCC) and a specific set of search terms. While WoSCC is a widely recognized, high-quality data source for bibliometric analysis, this reliance may limit the comprehensiveness of the retrieved literature, as studies indexed in other databases or published in languages other than English may not have been captured, potentially introducing selection bias. In addition, the present analysis was restricted to English-language publications indexed in the WoSCC, which may limit coverage of relevant studies from other databases or regions. Furthermore, bibliometric analysis is concerned with publication patterns and citation relationships rather than the methodological quality of individual studies. These limitations must be considered when interpreting the findings.

## 6. Conclusion

This study provides a comprehensive bibliometric overview of research on AI- and VR-enabled evaluation in medical education over the past decade. The findings suggest a discernible transition from conventional technology validation and skill-based assessment methodologies towards more quality-oriented, curriculum-integrated, and AI-enhanced evaluation approaches.

As medical education continues to evolve towards data-driven and competency-based models, the literature indicates that AI and VR may assume an increasingly significant role in shaping future evaluation practices. Further research is warranted to explore robust, transparent, and educationally grounded evaluation frameworks that can effectively support the assessment of complex medical competencies.

## Acknowledgments

We thank all researchers whose publications were included in this bibliometric analysis. The Education Reform Project 2024 at Chongqing Medical University (JY20240308) provided financial support for language editing and publication.

## Author contributions

**Conceptualization:** Xingxin Li, Na Wang, Yifan Zhang, Yun Luo, Xia Wu, Xi Huang.

**Data curation:** Xingxin Li.

**Formal analysis:** Xingxin Li.

**Funding acquisition:** Xi Huang.

**Investigation:** Xingxin Li.

**Methodology:** Xingxin Li.

**Project administration:** Xingxin Li.

**Resources:** Xingxin Li, Qianwei Lu, Di Li, Xi Huang.

**Software:** Xingxin Li.

**Supervision:** Xingxin Li, Yifan Zhang.

**Validation:** Xingxin Li, Zhengjun Guo.

**Visualization:** Xingxin Li.

**Writing** – **original draft:** Xingxin Li.

**Writing** – **review & editing:** Qianwei Lu, Zhengjun Guo, Yanni Hu, Xi Huang.
